# Acculturation, immigration status and cardiovascular risk factors among Portuguese immigrants to Luxembourg: findings from ORISCAV-LUX study

**DOI:** 10.1186/1471-2458-12-864

**Published:** 2012-10-11

**Authors:** Ala’a Alkerwi, Nicolas Sauvageot, Sybil Pagny, Jean Beissel, Charles Delagardelle, Marie-Lise Lair

**Affiliations:** 1 Centre de Recherche Public de la Santé, Centre d’Etudes en Santé, 1A rue Thomas Edison, Strassen, L-1445, Grand-Duchy of Luxembourg; 2 Centre Hospitalier de Luxembourg, 4, rue Barblé, Luxembourg, L-1210, Grand-Duchy of Luxembourg

**Keywords:** Cardiovascular risk factors, Acculturation, Portuguese immigrants, Cross-sectional study

## Abstract

**Background:**

No previous study has examined the prevalence of cardiovascular risk factors and explored the influence of immigration status and acculturation on overweight/obesity among the Portuguese immigrants to Luxembourg. Our objectives were to (1) compare the prevalence of cardiovascular risk factors between native Luxembourgers and Portuguese immigrants, (2) examine the relationship between immigrant generation status, proportion of life spent in Luxembourg and language proficiency or preference (as proxy variables of acculturation) and overweight/obesity among Portuguese immigrants, and (3) elucidate the role of underlying socioeconomic, behavioral and dietary factors in overweight/obesity differences among the two populations.

**Methods:**

Recent national cross-sectional data from ORISCAV-LUX survey 2007–2008, composed of 843 subjects were analyzed. Overweight/obesity was defined as body mass index (BMI) >25kg/m^2^. Acculturation score was measured by using immigrant generation status, proportion of life spent in Luxembourg, and language proficiency or preference. Univariable and multivariable logistic regression analyses were performed to examine the association between acculturation markers and overweight/obesity. Further, a series of successive models were fitted to explore the separated and added impact of potential mediators (socioeconomic status, physical activity, dietary factors) on overweight/obesity among Luxembourgers and Portuguese immigrants.

**Results:**

Compared to Luxembourgers, Portuguese immigrants of first and second generation were younger and currently employed. About 68% of first generation Portuguese had only primary school, and about 44% were living below poverty threshold. Although the cardiovascular risk factors were comparable, Portuguese immigrants were more frequently overweight and obese than Luxembourgers, even after age and gender standardization to the European population. Overweight/obesity was significantly higher among Portuguese of first generation compared to second generation (*P*=0.028). Although we observed a tendency of lower risk with higher acculturation, none of the acculturation markers, both individually and taken together as a score, was statistically significant after controlling for age and gender. Compared to Luxembourgers, odds of overweight/obesity were significantly higher among Portuguese immigrants, in unadjusted model 1 (*P*=0.043), in age and gender-adjusted model 2 (*P*<0.0001), in socioeconomic status adjusted model 3 (*P*= 0.01), in physical activity adjusted model 4 (*P*=0.007). However, this difference was attenuated and statistically disappeared after controlling for dietary factors (*P*=0.09).

**Conclusions:**

These findings address a lack of heterogeneity between Portuguese immigrants and Luxembourgers regarding hypertension, hyperlipidemia, diabetes mellitus, physical inactivity, and current cigarette smoking. However, Portuguese immigrants to Luxembourg were more likely to be overweight/obese than Luxembourgers participants. This risk may be explained by different dietary practice. An in-depth comparative assessment of dietary habits of Luxembourgers and Portuguese immigrants is warranted.

## Background

Cardiovascular diseases are the leading cause of mortality in Europe, accounting for more than 48% of all European deaths, with striking geographical variations [1]. Cigarette smoking, obesity, lipid disorders, elevated blood pressure and diabetes mellitus are not only associated with each other but also with high cardiovascular morbidity and mortality [2,3].

The Grand-Duchy of Luxembourg is a small country in the heart of Europe landlocked by Belgium, France and Germany, with a population of 511 840 inhabitants (official estimate, 2011) over an area of about 2600 km^2^. Cardiovascular mortality accounted for nearly one-third of death in 2010 [4]. According to a recent nationwide cross-sectional survey, the most predominant cardiovascular risk factors were dyslipidemia (69.9%), hypertension (34.5%), smoking (22.3%), obesity (20.9%), and diabetes 4.4% [5].

Distinctively, Luxembourg country is a multicultural society; about 43.2% of the people are foreign residents from over 150 different nationalities, among those, the Portuguese representing the major immigrant community (36.7%). According to the National Institute of Statistics (2011), Portuguese immigrants constitute 15.9% of the total Luxembourg population with a steady increase during the last decades [6]. Presently, about 80,000 people with Portuguese nationality reside in Luxembourg, following successive waves of post-war migration, associated with labor shortage. Evidently, the national health profile is significantly determined by the health of its immigrants.

Primary care physicians of Luxembourg have observed during the last decades that Portuguese immigrants to Luxembourg are more susceptible to develop cardiovascular diseases and are at higher risk of obesity, hypertension, lipid disorders and diabetes, compared to native Luxembourgers. However, this phenomenon observed by physicians needs to be supported by concrete evidence-based data. Several studies carried out in different countries worldwide, but mostly in American settings [7,8], suggest an influence of migration on the prevalence of cardiovascular risk factors. Cultural factors have been linked to poor health practices and risk of diabetes [9], hypertension [10], and obesity [11,12]. On the other hand, the literature suggests that migration may also have positive protective effects on obesity risk among lower income black immigrants to U.S. [13]. Diet quality of Tunisian migrants to the south of France partly explained their lower prevalence of diet-related chronic conditions compared to their native French peers [14]. On whole, health effects of acculturation vary by country of origin, health behaviors and outcome being studied; immigration and subsequent behavior changes may contribute either to the development or the decline of several cardiovascular disorders, such as obesity and diabetes [11,15].

Although Portuguese immigrants represent a large and growing segment of the Luxembourg population, it is unknown how cardiovascular risk factors differ between Portuguese immigrants and Luxembourgers. The particularity of Portuguese community constitutes an excellent opportunity to explore the implication of immigration on cardiovascular health in Luxembourg. The objectives of this study were to (1) compare the prevalence of cardiovascular risk factors between native Luxembourgers and Portuguese immigrants, (2) examine the relationship between immigrant generation status, proportion of life spent in Luxembourg and language proficiency or preference (as proxy variables of acculturation) and overweight/obesity among Portuguese immigrants, and (3) elucidate the role of underlying socioeconomic, behavioral and dietary factors in overweight/obesity differences among the two populations.

For these purposes, we used recent national cross-sectional data from ORISCAV-LUX survey (Observation of cardiovascular risk factors in Luxembourg, 2007–2008) [5]. Understanding the consequences of acculturation for obesity and its associated risk factors will have important implications which help to improve cardiovascular prevention for the whole population of Luxembourg.

## Methods

### Data sources

ORISCAV-LUX survey is the first nationwide population-based study carried out on a weighted random sample stratified according to age, sex and district (Luxembourg, Diekirch and Grevenmacher), of non-institutionalized adults, aged 18–69 years, residing in Luxembourg. The distribution of selected subjects in each stratum was proportional to their distribution in the source population. Further details concerning the aims, data collection, and sample representativeness have been reported elsewhere [5,16]. Briefly, information on demographic, socioeconomic and health-related characteristics was collected, with the help of trained personnel, via a self-administered questionnaire in French, German, Portuguese and English. The questionnaire was forward and backward-translated to French by independent certified translators and reviewed by multilingual staff. A total of 1432 subjects were recruited, of whom 191 were Portuguese immigrants and 652 Luxembourgers. Given the research objectives, only Portuguese participants were included in the analyses along with native Luxembourgers. The analyses were thus based on a sample of 843 subjects, excluding subjects with missing data on place of birth or length of residence in Luxembourg, and those from other different origins.

### Cardiovascular risk factors

Six cardiovascular risk factors of interest were studied: obesity/overweight, hypertension, hyperlipidemia, diabetes mellitus, physical inactivity, and current cigarette smoking, which were measured according to standard protocol procedures, as reported earlier [5].

Subjects with body mass index (BMI) >25kg/m^2^ were considered as overweight/obese [17]. BMI was calculated by dividing weight (kg) by the square of height (m), and coded as a dichotomous variable (obesity/overweight vs normal weight).

Data on diabetes, hyperlipidemia, and hypertension were all based on biological and objective measurements, as reported elsewhere [5,18]. Precisely, participants were classified as having elevated blood pressure if they reported taking anti-hypertensive medications and/or had SBP ≥ 140 mmHg and/or DBP ≥ 90 mmHg [19]. Participants were classified as diabetics if they reported taking anti-diabetic medications and/or had FPG ≥ 126 mg/dl [20]. Subjects with lipid disorder were defined as having at least one of the following anomalies: TC ≥ 190 mg/dl, TG ≥ 150 mg/dl, LDL-C ≥ 115 mg/dl, and HDL-C < 40 mg/dl for men and < 46 mg/dl for women [21], and/or taking hypo-lipid medications. These were coded as dichotomous variables (yes vs no) based on presence or absence of the relevant disease condition.

Smoking status was categorized as current smoker or non-smoker (which included former smokers and never smokers). Physical activity was self-reported using the International Physical Activity Questionnaire IPAQ [22], which categorizes the population into three levels: physically inactive, moderately active and active. The last 2 categories were regrouped into (active) versus (inactive).

### Acculturation variables

To examine the effect of acculturation on obesity differences between Portuguese immigrants, four measures were used: immigrant generation status, language proficiency or preference, proportion of life spent in Luxembourg, and acculturation score.

#### Immigrant generation status

The studied population was divided into 2 groups according to immigration status (Portuguese immigrants and native Luxembourgers). Portuguese immigrants were referred to both first and second generation of Portuguese immigrants to Luxembourg; participants who were born in Portugal were classified as “first generation”; participants who were born in Luxembourg and had one or both parents born in Portugal were classified as “second generation”. Generation is an important proxy measure that can provide important insight into the historical and geographical context, in addition to baseline cultural characteristics of the individual [23,24]. Those who were born in Luxembourg as were both parents were considered as native born “Luxembourgers”. A score of 1–3 was assigned to immigration status (1=Portuguese first generation, 2= Portuguese second generation, 3= Luxembourgers).

#### Language proficiency or preference

Language proficiency or preference during interview is a strong predictor of acculturation [25]. The participant indicated his/her preferred language to answer the self-administered questionnaire. The selection of either French or German, the two official languages in Luxembourg, represents dominance and proficiency with that language. A score of 0–1 was assigned to language proficiency or preference (0=Portuguese, 1= French or German).

#### Proportion of life spent in Luxembourg

Length of residency is an interesting indicator of exposure to the culture of the host country. As suggested by assimilation theory [26], the course of time and the exposure to the new culture can influence host satisfaction, which posits that immigrants will progressively converge into the mainstream. The number of years of residency in Luxembourg divided by age provides a fraction that indicates the proportion of an individual’s life spent in the second culture [27]. This continuous variable is ranged from 0.04 to maximum 1 (for those of second generation). Precisely, the minimum value means a subject of 55 years age who have been in Luxembourg since 2 years, then (2/55= 0.0366). The maximum value of the score indicates that a Portuguese subject spent all his life in Luxembourg (i.e., born in Luxembourg, so the period of residency=the age= 1).

#### Acculturation score

Similar to other research studies, a single summary acculturation score for each participant has been constructed, by summing the three individual indicators (immigrant generation status + language proficiency/preference + proportion of life spent in Luxembourg). It ranged from 1 (least acculturated) to 5 (most acculturated). The acculturation score has advantage that it takes into account simultaneously the characteristics which are often clustered within an individual. The combination of markers may give a more accurate representation of acculturation than each indicator independently [11].

### Other variables

Socio-demographic variables included age, gender, socioeconomic status (SES) as measured by level of education and income.

Concerning dietary habits, percentage of total daily energy intake of fat (%E), percentage of total daily energy intake of carbohydrates (%E), percentage of total daily energy intake of protein (%E) and fibers (grams per day) were estimated from the semi-quantitative self-administered Food Frequency questionnaire (FFQ) [28]. Full details concerning the calculation of variables have been published elsewhere [29].

### Ethical aspects

The present study was conducted according to the guidelines laid down in the Declaration of Helsinki and all procedures involving human subjects were approved by the National Research Ethics Committee *(Comité National d’Ethique de Recherche, CNER)* and the National Commission for Private Data Protection *(Commission Nationale pour la Protection des Données, CNPD)*. Written informed consent was obtained from all the subjects.

### Statistical analysis

Initially, participants’ characteristics by immigration status (in three categories) were compared and the P-values were presented. Each two groups were again compared and the difference was indicated whenever significant, by using the test of Kruskal-Wallis for non-normally distributed variables, an ANOVA for normally distributed variables, and *X*^2^ test for categorical variables.

Then, the six cardiovascular risk factors of interest (overweight/obesity, hypertension, dyslipidemia, diabetes mellitus, current cigarette smoking, and physical inactivity) were compared between the Luxembourgers and Portuguese immigrants. To facilitate a meaningful comparison between these two populations, with different age and gender composition, direct standardization method was applied to remove the effects of variation in age and gender structure using the European population as reference.

Further, univariable and multivariable logistic regression models were fitted to investigate the effect of acculturation, measured by three proxy indicators (immigrant generation status, length of residency/age and language proficiency or preference) on overweight/obesity among Portuguese immigrants. The effect of acculturation was tested by using each indicator individually and then by using the score which is the sum of the three proxy indicators. Portuguese of second generation and French/German were used as referents for immigrant generation status and language proficiency, respectively. A graphic presentation of the odds ratio and 95% confidence intervals are displayed in a Forest plot.

To explore the separated and added impact of potential mediators (SES, physical activity, dietary factors) on overweight/obesity among Luxembourgers and Portuguese immigrants, a series of successive models were fitted by adding, to the crude unadjusted Model 1, age and gender in Model 2; socioeconomic variables in Model 3; the physical activity in Model 4 and dietary factors in Model 5. Precisely, model 1=unadjusted; Model 2=adjusted for age and gender; Model 3=Model 2 plus SES (education in three categories; income in two categories); Model 4= Model 3 plus physical activity in three categories); Model 5= Model 4 plus dietary factors (total fat in percent Kilocalories, total carbohydrate in percent Kilocalories, total protein in percent Kilocalories, and total fiber in gram).

Results were considered significant at the 5% critical level (P<0·05). All statistical analyses were performed using PASW® for Windows® version 18.0 software (formerly SPSS Statistics Inc.)

## Results

### Characteristics of studied population according to immigration status

The characteristics of Luxembourgers and Portuguese Immigrants of first and second generation are presented in Table 1. Compared to Luxembourgers, Portuguese immigrants of first and second generation were significantly younger (*P*<0.0001) and currently employed. However, the educational attainment and income of first generation Portuguese were much lower than that of Luxembourgers and their counterpart of second generation. About 68% of first generation Portuguese had only primary school, and about 44% were living below poverty threshold. A remarkable economic status gradient between the two generations was also observed, in favor of the second generation immigrants.

**Table 1:** Characteristics of studied population according to immigration status, ORISCAV-LUX survey, 2007-2008

	**Portuguese 1**^**st**^**generation % (N)**	**Portuguese 2**^**nd**^**generation % (N)**	**Luxembourgers % (N)**	***P *value†**
n	169	22	652	
Age (years) (*n* 843) *	40.54 ±17	30.6 ± 7	47.71 ± 21	<0.0001^a, b, c^
Gender				0.092
Men (*n* 410)	55 (93)	59.1 (13)	46.6 (304)	
Women (*n* 433)	45 (76)	40.9 (9)	53.4 (348)	
Education level (%)				<0.0001^a, b^
Primary (*n* 245)	67.7 (113)	13.6 (3)	20 (129)	
Secondary (*n* 418)	28.7 (48)	54.5 (12)	55.6 (358)	
Tertiary (*n* 170)	3.6 (6)	31.8 (7)	24.4 (157)	
Economic status (%)				<0.0001^a, b^
below poverty threshold (*n* 144)	44.4 (68)	16.7 (3)	13.2 (73)	
above poverty threshold (*n* 578)	55.6 (85)	83.3 (15)	86.8 (478)	
Work status				<0.0001^a, b, c^
Employed (*n* 524)	74.6 (126)	81.8 (18)	58.4 (380)	
Non-employed (*n* 76)	7.1 (12)	18.2 (4)	9.2 (60)	
Housewife (*n* 107)	8.3 (14)	0 (0)	14.3 (93)	
Retired (*n* 135)	10.1 (17)	0 (0)	18.1 (118)	
Total dietary calories (Kcal) (*n* 796)*	2121.63 ± 1006	2307.06 ±1353	2274.18 ±1153	0.032^b^
Fat (% Kcal) (*n* 791)	35.43 ± 6.88	37.54 ± 4.93	39.13 ± 7.05	<0.0001^b^
Carbohydrates (% Kcal) (*n* 791)	43.84 ± 7.45	44.22 ± 6.21	41.95 ± 7.44	0.009^b^
Fibers (g/day) (*n* 796) *	22.3 ±12	20 ± 18	23.5 ± 13	0.207

Data are expressed as means ± SD, otherwise median (Inter-quartile interval) is indicated as*.

P values are from *X*^2^ tests for categorical variables, whereas ANOVA and Kruskal-Wallis were used for normally and non-normally distributed continuous variables, respectively.

† P-value indicates the comparison between the 3 groups.

a indicates that the P-value is significant when comparing Portuguese 1^st^ generation to Portuguese 2^nd^ generation.

b indicates that the P-value is significant when comparing Portuguese 1^st^ generation to Luxembourgers.

C indicates that the P-value is significant when comparing Portuguese 2^nd^ generation to Luxembourgers.

Luxembourgers reported considerably higher percentages of energy from fat than the Portuguese immigrants of first and second generation (*P*<0.0001), but with lower percentages of energy from carbohydrates intake (*P*=0.009).

### Prevalence of cardiovascular risk factors between Luxembourgers and Portuguese immigrants

**Figure 1:**
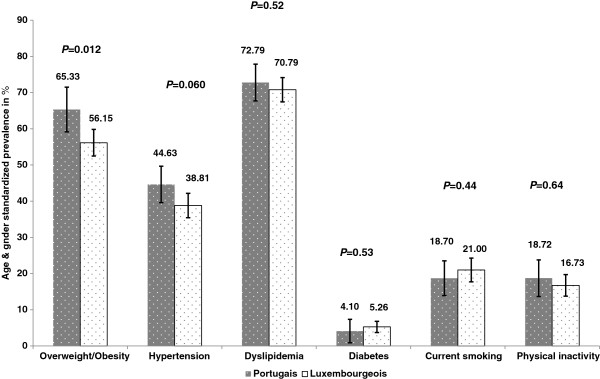
Comparison between Luxembourgers and Portuguese participants according to selected cardiovascular risk factors, ORISCAV-LUX survey, 2007–2008.

After age and gender standardization to European population, the distribution of cardiovascular risk factors was almost similar in both populations, with no significant difference as regards hypertension, lipid disorders, diabetes, smoking status and physical inactivity, except for overweight/obesity which was more common in Portuguese immigrants than Luxembourgers (65.33% vs 56.15%, respectively, *P*=0.012). The observed increase in the age- and gender-standardized prevalence of hypertension (44.6% vs 38.2%), dyslipidemia (72.8% vs 71.4%) and diabetes (4% vs 3%) among Portuguese immigrants was not statistically significant (Figure 1).

In light of these findings, we focused on examining relationships between the overweight/obesity status and the acculturation among Portuguese of first and second generation, by using our proxy variables of acculturation.

Portuguese was the preferred language for 70% of first generation Portuguese immigrants, whereas French or German were preferred for 80% of second generation. As expected, mean acculturation score was substantially higher in second than the first generation (3.86 ± 0.35 vs 1.75 ± 0.59, respectively). The overweight/obesity was significantly higher in first compared to second generation of Portuguese immigrants (*P*=0.028) (Table 2).

**Table 2:** Description of overweight/obesity and acculturation markers among Portuguese immigrants

	**Portuguese 1**^**st**^**generation (n=196)**	**Portuguese 2**^**nd**^**generation (n=22)**	*P *–value*
Language proficiency % (n)			<0.0001
Portuguese (*n* 121)	69.8 (118)	13.6 (3)	
French/German (*n* 70)	30.2 (51)	86.4 (19)	
Length of residency/age (means ± SD)	0.45 ± 0.23	1	
Acculturation score (means ± SD)	1.75 ± 0.59	3.86 ± 0.35	
Overweight/obesity % (n)	69 (116)	45.5 (10)	0.028

* P-value from *X*2 tests for categorical variables.

### Effect of generation, proportion of life spent in Luxembourg and language proficiency on overweight/obesity among Portuguese immigrants to Luxembourg

**Figure 2:**
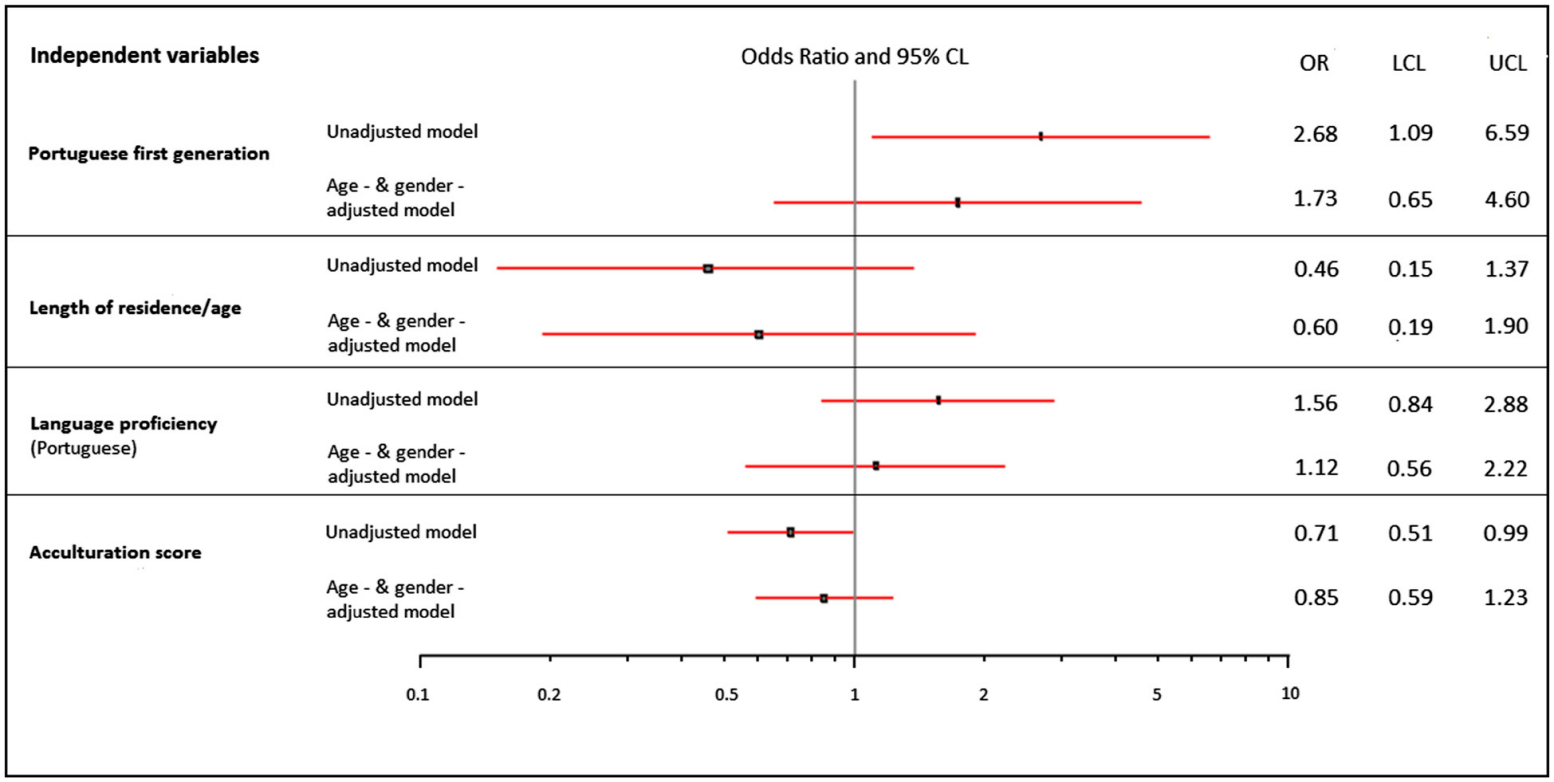
Odds ratio and 95% confidence intervals for the association of overweight/obesity and acculturation among Portuguese immigrants, measured individually and by score, ORISCAV-LUX survey, 2007–2008.

On examination of Portuguese immigrants’ population, first generation participants were at about two-fold odds to be overweighed/obese compared to second generation participants. This overweight/obesity-generation status association was only significant in the crude (unadjusted) model (OR=2.68; 95%CI: 1.09-6.56). In age and sex-adjusted model, the gradient of risk of overweight/obesity disappeared for the first compared to the second generation Portuguese immigrants (OR=1.73; 95%CI: 0.65-4.60). Likewise, the odds ratios for those who prefer Portuguese were higher in both crude (OR= 1.56; 95% CI: 0.84-2.88) and adjusted models (OR=1.12; 95% CI: 0.56-2.22) than those who prefer French/German, although this was not statistically significant. Regarding the proportion of life spent in Luxembourg, measured by the length of residency/age, higher fraction showed lower odds ratios in both crude (OR=0.46; 95% IC: 0.15-1.37) and adjusted model (OR= 0.60; 95% IC: 0.19- 1.90), but without sensible statistical significance. With regard to acculturation score, the magnitude and the significance of effect on overweight/obesity prevalence was slightly protective in the crude model, but this effect disappeared in the adjusted model (Figure 2).

In the next step, we focused on the role of underlying socioeconomic, behavioral and dietary factors in overweight/obesity differences among the two populations (Portuguese immigrants and Luxembourgers).

### Role of underlying socioeconomic, behavioral and dietary factors in overweight/obesity differences between Luxembourgers and Portuguese immigrants

Given that only overweight/obesity was significantly higher in Portuguese immigrants compared to Luxembourgers, we ran a series of models to examine the extent to which SES, physical activity and dietary factors explain this disparity. We found that odds of being overweight/obese were significantly higher among Portuguese immigrants as compared to Luxembourgers, in unadjusted model 1 (*P*=0.043), and in age and gender-adjusted model 2 (*P*<0.0001). The difference was still significant, by adding socioeconomic status (*P*= 0.01 in model 3), physical activity (*P*=0.007 in model 4), in the sense that Portuguese immigrants were at about double-fold odds to be overweight/obese compared to Luxembourgers. However, this difference was attenuated and statistically disappeared after controlling for dietary factors (*P*=0.09) (Table 3).

**Table 3:** Simple and adjusted logistic regression models of prevalent overweight/obesity among Luxembourgers and Portuguese immigrants, ORISCAV-LUX survey, 2007-2008

	**Odds ratio (95% CI)**		**P value**
	**Luxembourgers (*n *652)**	**Portuguese immigrants (*n *191)**	
Model 1 unadjusted	Referent	1.42 (1.01 - 1.99)	0.043
Model 2 adjusted for age and sex	Referent	2.08 (1.42 - 3.03)	<0.0001
Model 3 (Model 2+ SES)	Referent	1.83 (1.16 - 2.90)	0.01
Model 4 (Model 3+ physical activity)	Referent	1.91 (1.19 - 3.08)	0.007
Model 5 (Model 4 + dietary factors)	Referent	1.57 (0.93 - 2.65)	0.09

Model 1=unadjusted; Model 2=adjusted for age and gender; Model 3=Model 2 plus SES (education in three categories; income in two categories); Model 4= Model 3 plus physical activity in three categories); Model 5= Model 4 plus dietary factors (total fat in percent Kilocalories, total carbohydrate in percent Kilocalories, total protein in percent Kilocalories, and total fiber in gram).

## Discussion

In line with traditional countries of immigration, such as Australia, Canada and the United States, the issue of migrant health receives increasing attention in Europe [30], and particularly in Luxembourg. Accurate data on the health of Portuguese migrants are an essential pre-condition for providing appropriate and accessible health services to this population group.

In contrast to the widespread opinion among the general practitioners, this analysis of a large national sample documented, for the first time, an absence of heterogeneity between Luxembourgers and Portuguese immigrants to Luxembourg, concerning the most important preventable and potentially modifiable cardiovascular risk factors, except the higher overweight/obesity among the Portuguese. As in most European countries, the organization of Luxembourg’s health care services ensures equal access and utilization to different immigrant groups. In addition, a number of measures were already implemented by policy-makers to improve access of immigrants to health services. These include overcoming language barriers and poor communication by providing interpretation and regular Portuguese translation of official documents. The translated documents help publicizing health education messages to immigrants. Therefore, our findings may reflect a national parity in health and equal opportunities to care access.

Prior studies have suggested mixed evidence concerning the relationship between acculturation, lifestyle behaviors, and cardiovascular risk factors among immigrants [7,8]. Several studies suggest that acculturation is associated with some positive healthy behaviors, such as leisure-time physical activity [31], whereas others suggest that the positive health behaviors decline with acculturation [32,33]. It is noteworthy that similar prior studies were largely carried out in US, few studies carried out in Europe, and even none in Luxemburg.

Luxembourg’s immigration rate rose sharply in the course of the last century, with a noticeably important influx of Portuguese immigrants during the last 40 years. In contrast to the tendency of other minorities, a large majority of Portuguese immigrants seem to keep their original culture. The geographical proximity to their country of origin allows for frequent contact and travel. Many resident Portuguese continue to speak Portuguese and are closely tied to their cultural roots; however, many do learn the official languages of Luxembourg (French and German, beside Luxembourgish), integrate the new culture, and thus become bicultural and multilingual to different extents. The distinct cultural particularity of Portuguese immigrants to Luxembourg provided the opportunity to examine the overweight/obesity-acculturation relationship among Portuguese of first and second generation. For this objective, we considered three proxy indicators including proportion of life spent in Luxembourg, language proficiency or preference, and immigrant generation status. Although none of the acculturation markers, both individually and taken together as a score of acculturation, were statistically significant after controlling for age and gender, we observe a tendency of lower risk with higher acculturation. The absence of statistically sensible effect may be related to low sample size of Portuguese of second generation. In fact, the selected ORISCAV-LUX sample was representative of the source population with respect to nationality, however, the participation rate was lower in Portuguese residents (13.62%, *n* 195) as compared to Luxembourgers (62.08%, *n* 889) [16]. Positively, the breadths of confidence intervals were not excessively wide-ranging, particularly for the acculturation score, which gives a more accurate representation of acculturation than each indicator alone. These confidence intervals provide an ‘estimate interval’, which gives us a measure of ‘precision’ or ‘confidence’ around our point estimate; wide confidence intervals indicate lack of precision [34]. Therefore, we globally concluded that the most acculturated Portuguese immigrants seemed to be protected and less susceptible to be overweighed/obese than the least acculturated. The absence of levelheaded association is probably because the effect size (culture) is too small to get a statistically significant result, i.e., although may exist, it couldn’t be detected by the actual sample size.

Another potential explanation of our finding may be related to the “healthy migrant effect phenomenon” [35]. Although immigrants face particular health challenges and are vulnerable to a number of threats to their physical and mental health, they are often healthy and enjoy a good health to work, notably in case of successive labor Portuguese migration.

In addition, the prevalence of main cardiovascular risk factors, in particular overweight/obesity are highly elevated both in Portugal [36] and in Luxembourg [5]. This homogeneity between the two populations may limit the ability to detect a discriminative effect of acculturation.

Overweight/obesity is a national public health problem. According to previous ORISCAV-LUX survey findings, more than 50% of adults’ residents in Luxembourg were affected, the prevalence increased with age, and varied by country of birth [5]. In this study, we documented a higher prevalence of overweight/obesity among Portuguese immigrants compared to Luxembourgish participants. About two-fold odds associated with migration status (Luxembourgers versus Portuguese immigrants) remained after adjustment for age, gender, and socioeconomic status. Further adjustment for physical activity did not significantly change this association, but adjustment for dietary factors attenuated the relationship between migration status and overweight/obesity. This finding suggests that diet may be an important contributor to the risk of overweight/obesity in Portuguese immigrants and dietary practice may explain the high likelihood of overweight/obesity among Portuguese subjects.

Owing to the total lack of knowledge concerning the health status of Portuguese immigrants to Luxembourg, the current study constitutes the first exploratory study to address the prevalence of cardiovascular risk factors between Luxembourgers and Portuguese immigrants and thus fill in the gap knowledge as regards cardiovascular health and immigration status in Luxembourg. It confers a valuable baseline evidence for health professionals, hospital managers and public health decision-makers to obtain more knowledge on Portuguese migrants’ health.

Despite the unique and innovative findings which are based on recent data (2007–2008), the study has however several limitations. It is possible that only healthier Portuguese have participated and hence little variability was detected between Portuguese and Luxembourgers, as regards the cardiovascular risk factors. Despite the observed protective tendency of acculturation, the small sample size of second generation might limit the statistical power to detect a sensible meaningful association between acculturation and overweight/obesity. However, low response rates and small sample sizes are typical limitations in most immigrant data collected from the population-based surveys, which may partly explain the important heterogeneity in the literature regarding the association between acculturation, health behaviors and chronic disease prevalence [9].

The ORISCAV-LUX survey was primarily not designed to examine migration and acculturation effect among minorities. However, it provided information on four proxy measures of acculturation [33]: language proficiency or preference, length of residency/age (proportion of life lived in Luxembourg), immigrant’s generation and country of birth. The majority of studies on health and acculturation use different measures of acculturation, and this variation may account for different results across the studies. Although the proxy measures have been widely used in similar studies [23,24,27], these variables do not fully capture the complex process of acculturation and its health effects [11]. Acculturation is an indication of the cultural change of minority individuals to the majority culture. Across literature, the relationship between acculturation and health status in immigrant population vary widely according to the construct used, immigrant population, and outcomes of interest. It has been demonstrated that when thoroughful assessments of acculturation are unfeasible or unavailable, shorter proxy measures can be useful and constitute suitable substitutes to assess acculturation, with several advantages: simplicity of assessment, feasibility of collection in large health surveys, and limited respondent burden [37].

Given the high prevalence of cardiovascular risk factors among our growing multicultural society in Luxembourg, a future larger scale cross-cultural study is warranted.

## Conclusions

In conclusion, the cross-sectional analysis, adjusted for age, gender, socioeconomic status and physical activity, revealed that Portuguese immigrants to Luxembourg were generally more likely to be overweight/obese than native Luxembourger participants. This risk may be explained by the dietary factors. From public health standpoint, these findings are important in delineating the groups at risk of overweight, given the substantial increase in overweight and obesity-associated morbidity and mortality.

### Perspectives

In order to design appropriate public health policies and health promotion interventions, greater consideration of the cultural environment may be warranted [38]. In this regard, an in-depth cross-cultural analysis of dietary habits between Luxembourger and Portuguese participants will increase our understanding of the potential influence of cultural environments on diet composition [39]. 

Another challenging avenue of future research is to compare the cardiovascular risk of Portuguese immigrants to that of those living in Portugal. Such cross-nation comparison is relevant for etiological purposes, and allows demonstrating and quantifying the contribution of different types of factors, including genetic factors, early living conditions, behavioral factors, health and integration policies, and their interactions on health status of immigrants.

## Competing interests

The authors declare that they have no competing interests.

## Authors' contributions

AA was involved in the conception and design of the ORISCAV-LUX survey, coordinated the field data collection, conceived the present research, contributed to data analyses and drafted the manuscript. NS and SP performed the statistical analyses and discussion of the results. JB, CDG Cardiologists contributed to the critical revision of the manuscript. M-LL was involved in the instigation of the ORISCAV-LUX study and critical revision of the manuscript. All authors reviewed and approved the final version of the manuscript.

## Pre-publication history

The pre-publication history for this paper can be accessed here:

http://www.biomedcentral.com/1471-2458/12/864/prepub
